# Effects of porous structure on the deformation failure mechanism of cement sheaths for wellbores

**DOI:** 10.1038/s41598-023-35398-9

**Published:** 2023-06-27

**Authors:** Yongming Yang, Xiwen Li, Mengke Sun, Yang Ju

**Affiliations:** 1 School of Mechanical & Civil Engineering, China University of Mining and Technology, D11 Xueyuan Road, Beijing, 100083 China; 2 State Key Laboratory of Coal Resources and Safe Mining, China University of Mining and Technology at Beijing, Beijing, 100083 China; 3 State Key Laboratory for Geomechanics & Deep Underground Engineering, China University of Mining and Technology, Xuzhou, 221116 China

**Keywords:** Natural gas, Petrol

## Abstract

The influence and mechanism of porous structure on the deformation failure of cement sheaths under hydraulic pressure is still unclear. To solve this problem, a net slurry cement sheath and a liquid silicon cement sheath were prepared by using a cement material and a liquid silicon suspension. The distributions of the pore radius and spatial location were analyzed using computed tomography scanning and statistics to obtain their probability density distribution functions. Based on the distribution functions, the single-layer and double-layer porous reconstruction models of the net slurry cement sheath and liquid silicon cement sheath were constructed using a FLAC 3D program. A series of numerical simulations were conducted to study the deformation failure of the cement sheaths under in situ stress and hydraulic pressure. The effects of the porous and double-layer structures on the breakdown pressure, plastic failure zone, radial deformation, and stress distribution of the cement sheaths were analyzed. As a result, the mechanisms for the influence of the porous and double-layer structures on the failure mode, failure path, and interaction between the cement sheath and metal casing were revealed. The results of this research provide a theoretical basis for an in-depth understanding of the failure mechanisms of porous cement sheaths.

## Introduction

Hydraulic fracturing is a common stimulation method for exploiting unconventional oil and gas resources, such as coalbed methane, shale gas, and shale oil, in low-permeability fields^1–6^. The safety of metal casings in fractured wells is crucial for implementing hydraulic fracturing technology in the field^7,8^. To ensure the safety and stability of a metal casing, cement mud should be injected between the metal casing and the wellbore to form a cement sheath. The cement sheath can effectively prevent corrosion, deformation, and failure of the metal casing^9–12^. Failure of the cement sheath can cause annular pressure and oil and gas interflow in the strata, resulting in a poor stimulation effect for oil and gas recovery. Moreover, it can cause deformation of the metal casing and fracturing well collapse, which can result in hydraulic fracturing process failure and very large economic losses^13–16^. Studies have found that the cement type, stratum conditions, fracturing methods, and temperature environment significantly affect the safety of cement sheaths^17–26^. Therefore, ensuring the integrity and safety of cement sheaths is crucial for the safe exploitation of unconventional oil and gas resources.

As early as the 1970s, researchers found that stress effects could affect the integrity of cement sheaths^27–29^. In the 1990s, a double-layer concentric casing simulation device was established to study the influence of stress on the tightness of a cement sheath under different temperatures and pressures^30^. The results showed that radial cracks in the cement sheath caused by the casing pressure considerably influenced cement ring failure. Furthermore, a mathematical model of the coupling of temperature and stress on a cement sheath under nonuniform in situ stress conditions was established. The effects of changes in casing temperature and pressure on stress magnitude and distribution in a cement sheath under nonuniform in situ stress were studied^31^. Subsequently, the Mohr‒Coulomb failure criterion was used to evaluate the integrity of a cement sheath based on cement mechanical properties, casing pressure, and initial temperature^32^. To explore the failure mechanism of cement sheaths and establish preventive measures, a set of integrity test devices for cement sheaths was developed, and experimental research on cement sheath integrity was conducted under concentric and eccentric conditions. Available research indicates that reducing the elastic modulus of a cement sheath can decrease stress and prevent its failure^33^. Some scholars added rock asphalt particles modified by plasma technology into cement sheaths and tested their mechanical parameters. They found that rock asphalt particles could effectively strengthen a viscoelastic cement sheath and increase the frictional force between cracks, thereby improving the impact resistance and plasticity of the cement sheath^34^.

In summary, these studies have played a positive role in deepening the understanding of the deformation and failure mechanisms of cement sheaths and improving their integrity and stability. However, cement is a heterogeneous material with numerous micropores that can considerably influence the deformation and failure of cement sheaths under conditions of in situ stress and hydraulic fracturing^35–38^. At present, research on cement sheath failure mainly focuses on the material characteristics of cement sheaths, crack geometry, and damage behavior^39–47^. Few studies have been conducted on the distributions of porous structures in cement sheaths and their influence on the stress distribution and deformation of cement sheaths, especially their failure modes and failure paths. The mechanisms by which porous structure influences the deformation failure of cement sheaths are ill understood. Therefore, it is necessary to explore the effects of porous structure on the stress distributions, deformation characteristics, failure modes, and failure paths of cement sheaths and revealing their deformation and failure mechanisms. This can effectively prevent damage of cement sheaths and improve their integrity to reduce the very large economic loss caused by cement sheath failure in oil and gas exploitation.

**Figure 1:**
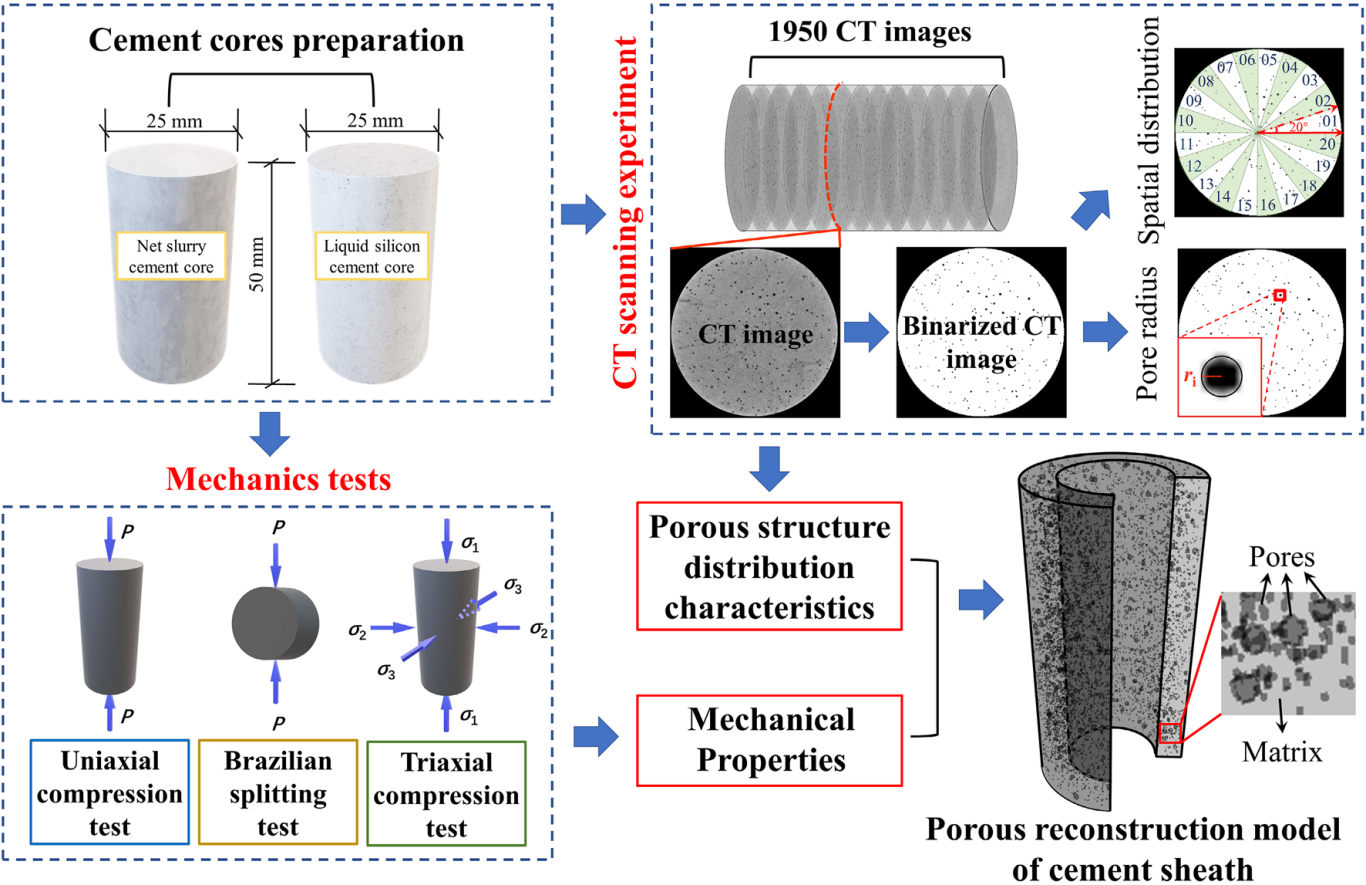
Reconstruction of porous models of cement sheaths.

To solve the abovementioned problems, two types of cement sheaths with different porosities were prepared: a net slurry cement sheath and a liquid silicon cement sheath. The distribution characteristics of the micropores of the two types of cement sheaths were analyzed using CT scanning experiments. A FLAC 3D program was developed in this laboratory and applied to construct porous models of the net slurry cement sheath and liquid silicon cement sheath to simulate their deformation and failure process under the combined action of in situ stress and hydraulic pressure. The reconstruction of porous models of cement sheaths is shown in Fig. 1. The effects of the porosity on the breakdown pressure, stress distribution, deformation, failure mode, and failure path of the cement sheaths were studied. Models of single-layer cement sheaths and double-layer cement sheaths were employed to explore the effect of the double-layer structure on failure and the mechanism of the interaction between the cement sheaths and metal casing.

## Methodology

### Preparation of cylindrical cores

**Figure 2:**
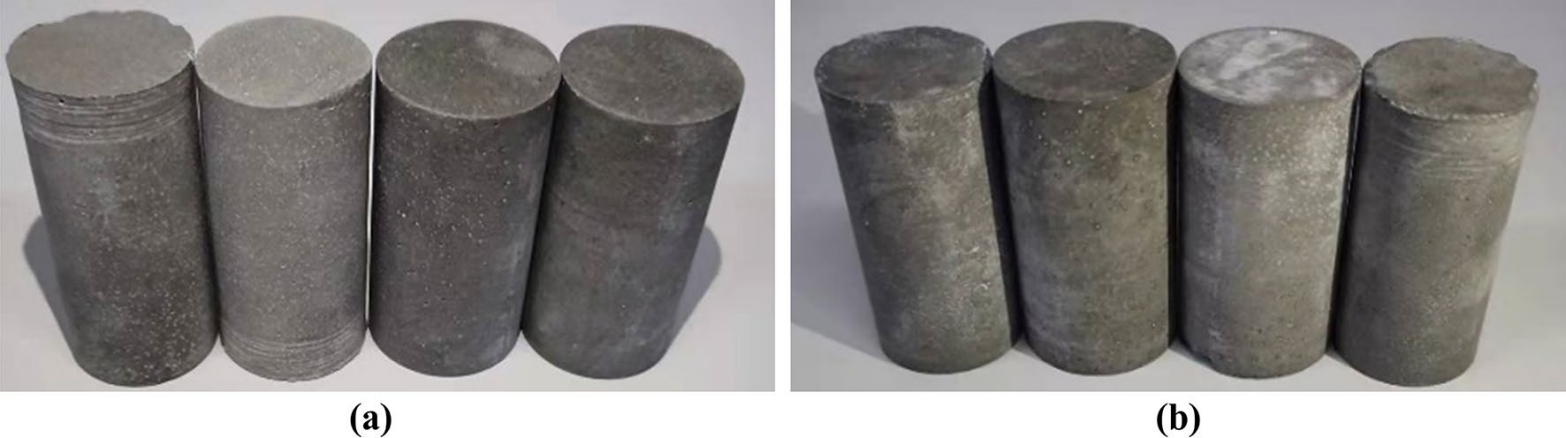
Cylindrical cores of cement sheaths. (**a**) Net slurry cement; (**b**) Liquid silicon cement.

In this study, two types of cylindrical cores of cements with different porosities were fabricated: net slurry cement cores and liquid silicon cement cores. The sizes of the cylindrical cores were *ϕ*25 mm × 50 mm. The materials included cement, quartz sand, water reducer, water, and liquid silicon suspension. The ratios of materials were based on the engineering ratios on site, as shown in Table 1. The specific preparation process was as follows: First, materials were weighed according to the material ratio. Second, appropriate weights of cement and quartz sand were placed into a mixer and stirred for 3 min. Subsequently, water and liquid silicon suspensions with appropriate weights were added to the mixer and stirred again for 2 min. Third, the mixture was poured into a cylindrical mold, placed on a vibrating table and vibrated for 2 min. The cylindrical core from the mold was removed after 24 h. Finally, the cylindrical core was placed in a 90 ℃ curing box for 72 h. The prepared cylindrical cores are shown in Fig. 2. The production process and curing time were the same for all cylindrical cores, which ensured the consistency of the distributions of the pores and mechanical properties of specimens of each type of cement core.

**Table 1:** Material ratios of cements.

	Water cement ratio	Cement/kg	Quartz sand /kg	Water reducer/ml	Water content of water reducer/ml	Water/ml	Liquid silicon suspension/ml
Net slurry cement	0.14	0.35	0.35	6.25	4.22	42	2.1
Liquid silicon cement	0.14	0.35	0.35	6.25	4.22	42	0

### Mechanical properties of cement sheaths

**Figure 3:**
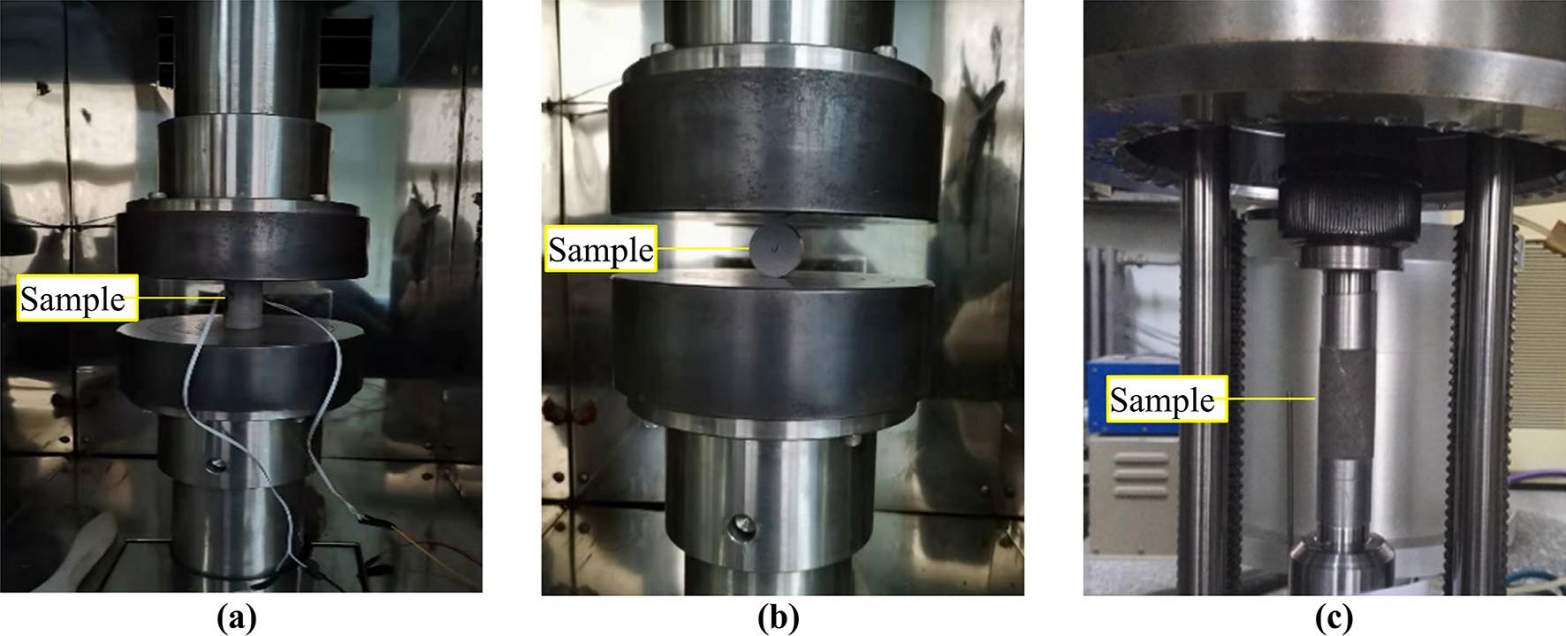
Photographs of experiments. (**a**) Uniaxial compression test; (**b**) Brazilian splitting test; (**c**) Triaxial compression test.

**Figure 4:**
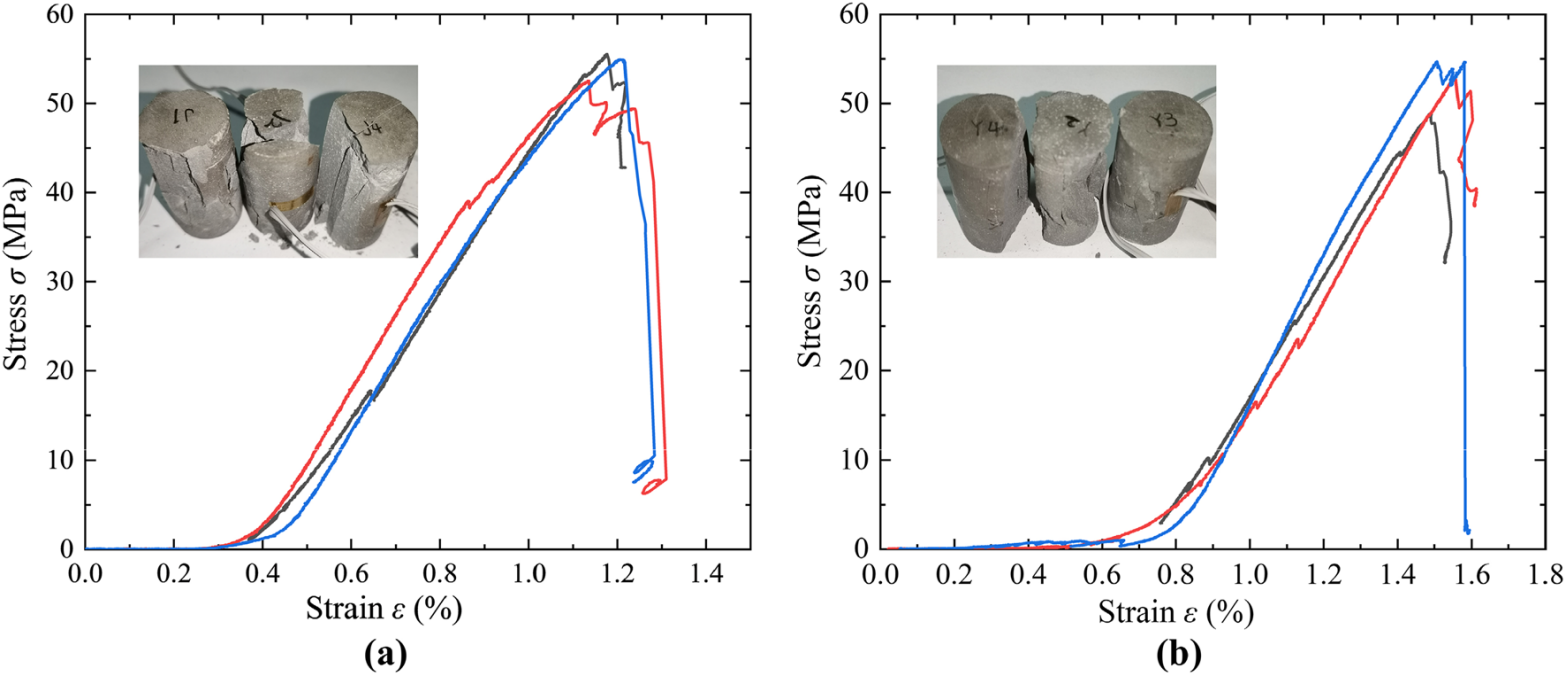
Stress–strain curves of uniaxial compression. (**a**) Net slurry cement; (**b**) Liquid silicon cement.

Uniaxial compression tests, Brazilian splitting tests, and triaxial compression tests were performed to obtain the mechanical parameters of the net slurry and liquid silicon cement cores. The experimental results for the mechanical properties of cement sheaths were used as the material properties of the numerical models to ensure the accuracy of the results from the numerical simulations. The loading rate for the uniaxial compression test was 0.5 mm/min. Three groups of experiments were conducted for each cement type. The experimental results were averaged to obtain the uniaxial compressive strength, elastic modulus, and Poisson ratio. The loading rate in the Brazilian splitting experiment was 0.1 mm/min. Three groups of experiments were also conducted for each type of cement, and the experimental results were averaged to obtain the tensile strength. Triaxial compression tests were performed using a triaxial servo testing machine with confining pressures of 5, 15, and 25 MPa to test the cohesion force and internal friction angle. The experimental results are listed in Table 2. Figure 3 shows photographs of the three types of experiments, and Fig. 4 shows the stress‒strain curves of uniaxial compression.

**Table 2:** Experimental results for the mechanical parameters of cements.

	Tensile strength/MPa	Compressive strength /MPa	Elasticity modulus/GPa	Poisson’s ratio	Cohesion force/MPa	Internal friction angle/°
Net slurry cement	2.0	53.78	4.59	0.16	5.6	30
Liquid silicon cement	4.8	54.31	3.56	0.15	7.8	35

### Distributions of porous structures in cement sheaths

**Figure 5:**
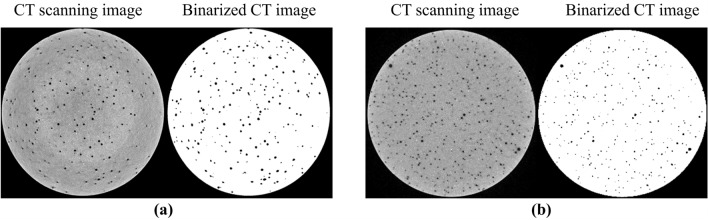
Binarized CT images of the porous structure. (**a**) Net slurry cement; (**b**) Liquid silicon cement.

To analyze the distributions of the porous structures of the net slurry and liquid silicon cement sheaths, CT scanning experiments were performed by using an industrial CT scanning system. Consequently, CT scanning images of their porous structure were obtained. A total of 1950 CT images of each type of cement core were obtained using volume scanning with a scanning interval of 25.6 µm. The CT images were preprocessed to improve quality. First, a Gaussian filter and the median filter algorithm were used to process the noise points and ring artifacts of the CT images. Second, a threshold segmentation method was used to binarize the CT images. The binarized CT images of the porous structures are shown in Fig. 5.

Using the program developed in this laboratory, the porosities of all binarized CT images of the net slurry cement sheath and liquid silicon cement sheath were calculated, and the average value was obtained. The average value represents the porosity of the cement sheath. The calculated results show that the porosity of the net slurry cement sheath was 1.25%, and that of the liquid silicon cement sheath was 0.86%.

### Distributions of spatial locations of pores

**Figure 6:**
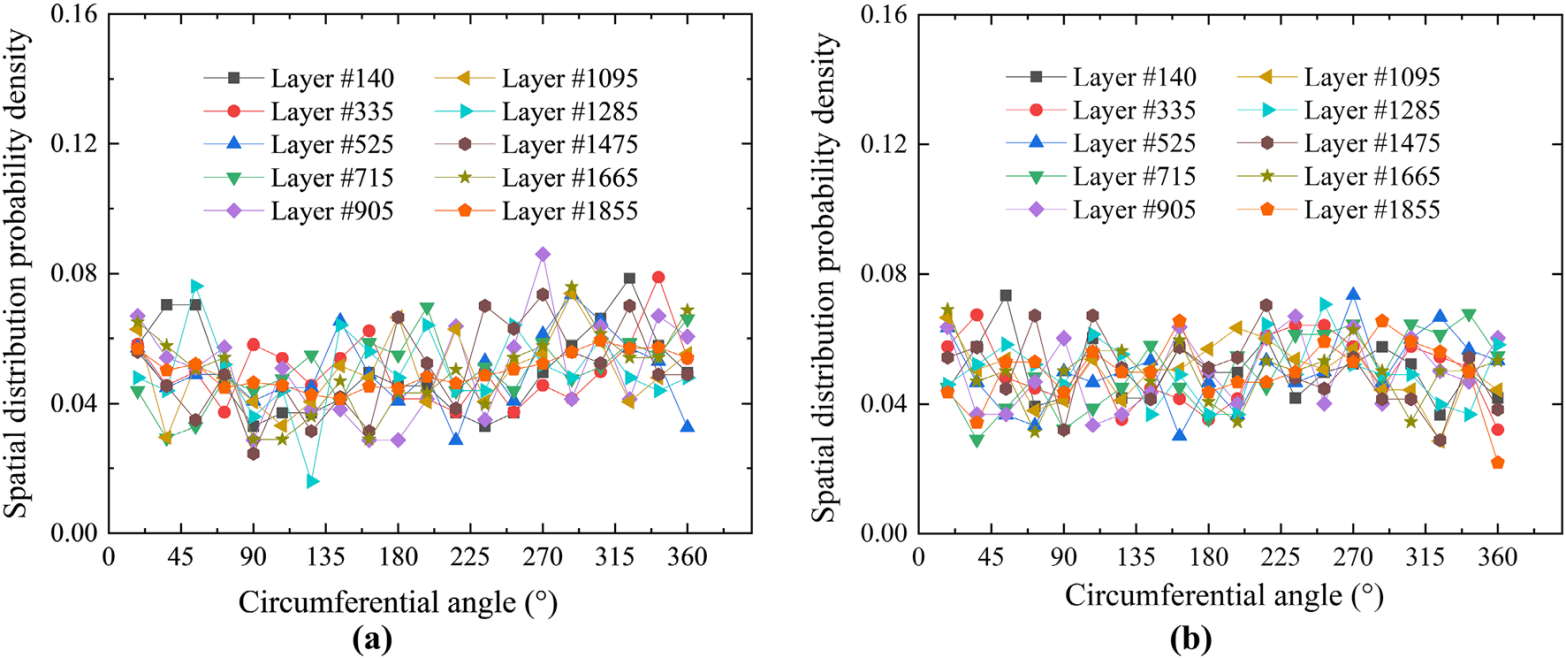
Distributions of spatial locations of the pores. (**a**) Net slurry cement; (**b**) Liquid silicon cement.

In this study, the distributions of the spatial locations of the pores of the clean slurry cement sheath and liquid silicon cement sheath were analyzed, and the distribution curves were obtained. The specific analysis process was as follows: Ten CT images were selected as representative layers from 1950 CT images of each type of cement sheath, numbered 140, 335, 525, 715, 905, 1095, 1285, 1475, 1665, and 1855. The CT images of all representative layers were evenly divided into 20 equal parts along the circumferential direction, and the number and probability density of pores in each part of each representative layer were calculated using a program developed in this laboratory. The probability density curves of the pores of every representative layer along the circumferential distribution were obtained, as shown in Fig. 6.

For both the net slurry and liquid silicon cement sheaths, the probability density of the pore distribution along the circumferential direction of all representative layers was concentrated in the range of 4–8%, which was approximately constant. This indicates that the spatial locations of the pores were approximately uniformly distributed along the circumferential direction.

### Distribution characteristics of pore radius

**Figure 7:**
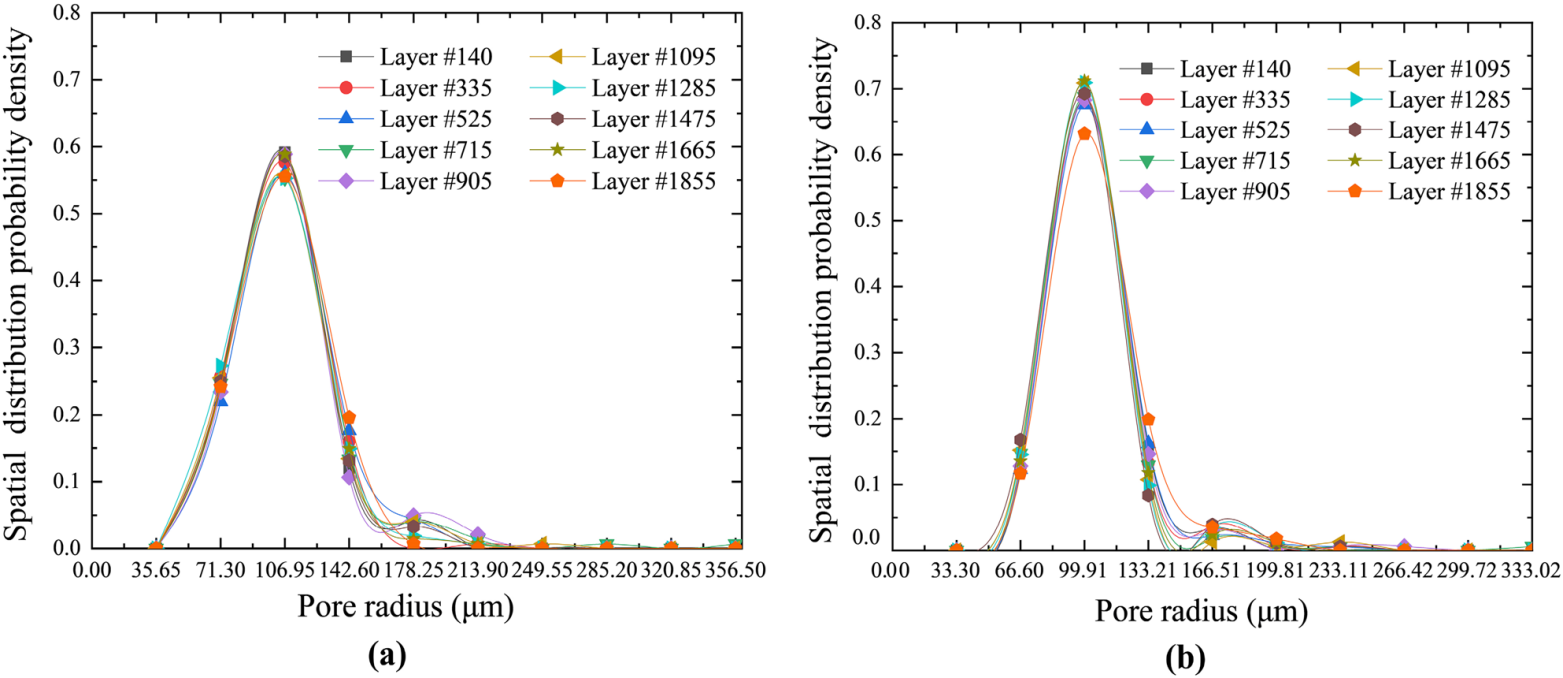
Probability density distributions of the pore radius. (**a**) Net slurry cement; (**b**) Liquid silicon cement.

Analyzing the binarized CT images of the porous structure showed that the radial distribution of the pores the net slurry cement sheath was 35.65–356.48 μm and that of the liquid silicon cement sheath was 33.3–333.02 μm. According to the upper and lower limits of the distribution range of each cement sheath type, the distribution range of the pore radius was divided into 10 equal parts. The number and probability density of pores in each part were calculated using a program developed in this laboratory. Figure 7 shows the probability density distributions of the pore radii of all representative layers.

Figure 7 shows that the probability density distributions of the pore radii of the net slurry cement sheath and liquid silicon cement sheath were Gaussian. The maximum pore radius of the net slurry cement sheath was 356.48 μm, and the minimum pore radius was 35.65 μm, while those of the liquid silicon cement sheath were 333.02 μm and 33.3 μm, respectively. Data for the net slurry cement sheath were larger than those of the liquid silicon cement sheath. Additionally, the radii of the pores with the highest proportion in the net slurry cement sheath were approximately 106.95 μm, and the corresponding pore area was 3.6 × 104 μm^2^, while that in the liquid silica cement sheath was approximately 99.91 μm, and its area was 3.1 × 104 μm^2^, approximately 15% smaller than that of the net slurry cement sheath. The liquid silicon cement sheath had a smaller pore radius than the net slurry cement sheath. This indicated that adding an appropriate amount of liquid silicon suspension in the mixture of material could effectively reduce the pore size and porosity of the cement sheath and increase its compactness. The probability density distribution functions of the pore radii of the two types of cement sheaths could be expressed as shown in Eq. (1).

The pore radius probability density distribution function:

1 $$f(r) = L_{0} + \left( {\frac{A}{{B\sqrt {\frac{\pi }{2}} }}} \right)e^{{ - 2\left( {\frac{{r - r_{0} }}{B}} \right)^{2} }}$$

where *L*_0_, *r*_0_, *A*, and *B* are the statistical parameters and $r$ is the pore radius. Moreover, $f(r)$ is the number and probability density of pores with radius $r$. According to the experimental conditions in this study, the values of the four statistical parameters of the net slurry cement sheath were 0.0032, 102.73, 47.64, and 34.5, and those of the liquid silicon cement sheath were 0.0057, 99.51, 36.32, and 31.12.

### Pore formation in the reconstruction model

The pores were treated as spheres in the reconstructed model. Let the volume be $V$ and the porosity be $\rho_{v}$ of the reconstruction model of the cement sheath.

According to the probability density distribution function of the pore radius, the number $n(r_{i} )$ and total volume $V(r_{i} )$ of pores corresponding to radius $r_{i}$ can be calculated. The formulas are shown in Eqs. (2)–(3):

2 $$n(r_{i} ) = \frac{{V\rho_{v} }}{{\sum\nolimits_{j} {\left( {f(r_{j} )\frac{4}{3}\pi r_{j}^{3} } \right)} }}f(r_{i} ),$$

where $r_{i}$ and $r_{j}$ are the pore radii. $V$ and $\rho_{v}$ are the volume and porosity of the reconstruction model of the cement sheath. $n(r_{i} )$ and $f(r_{i} )$ are the number and probability density of pores with radius $r_{i}$. Moreover, $f(r_{j} )$ is the number and probability density of pores with radius $r_{j}$.

3 $$V(r_{i} ) = n(r_{i} )\frac{4}{3}\pi r_{i}^{3} ,$$

where $V(r_{i} )$ is the total volume of pores with radius $r_{i}$.

A hexahedral mesh was used as the porous reconstruction model. According to Eq. (4), the number of meshes occupied by the pores with radius $r_{i}$, $N(r_{i} )$, can be calculated:

4 $$N(r_{i} ) = \frac{{V(r_{i} )}}{{l^{3} }},$$

where $l$ denotes the side length of the hexahedral mesh.

According to the aforementioned analysis, the spatial locations of the pores satisfied a uniform distribution, and the probability densities of the pore radii satisfied a Gaussian distribution. According to the probability densities of different pore radii, the number of each level of pores in the constructed model was calculated. Using the Monte Carlo method and the program developed in this laboratory, three sets of uniformly distributed random numbers were generated in turn as the coordinates of the pores. Subsequently, data integration was performed to obtain the coordinates of all pores for the target model. The pores in the reconstruction model were determined by combining the mesh number of pores and their spatial coordinates.

### Porous model reconstruction

According to the field data, the geometric dimensions of the cement sheath and metal casing with a depth of 1200 m are listed in Table 3.

**Table 3:** Geometric dimensions of the cement sheath and metal casing.

Depth/m	Inside radius of casing/mm	Outside radius of casing/mm	Inside radius of inner layer cement sheath/mm	Outside radius of outer layer cement sheath/mm
1200	111.2	122.25	69.85	157.66

**Figure 8:**
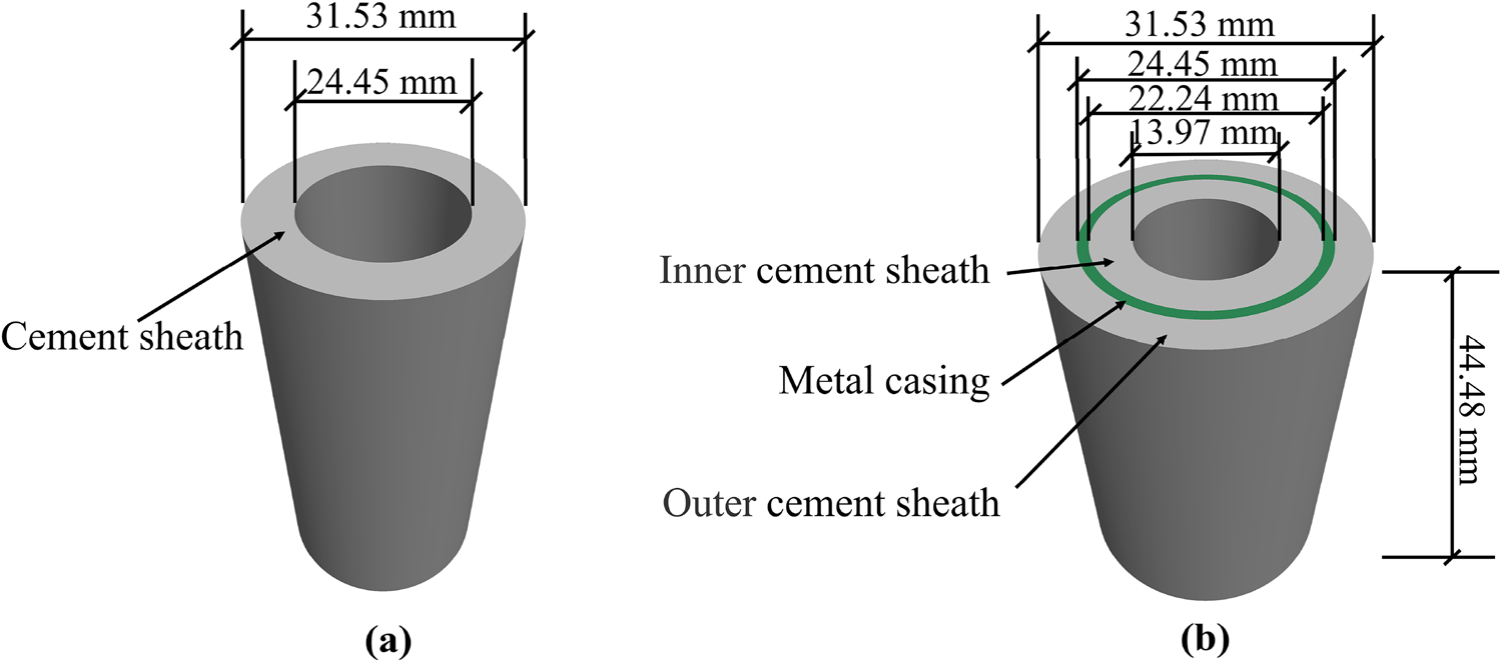
Geometric parameters of the cement sheath and metal casing. (** a**) Single-layer model; (**b**) Double-layer model.

The pore size was very small compared to the cement sheath size, with a difference of five orders of magnitude. To accurately reproduce the micropores in the reconstruction model, the mesh of the reconstruction model was finely divided. As a result, the number of meshes and the computational scale increased sharply. This complicated numerical calculations or even made them impossible. To solve this problem, the geometric dimensions of the cement sheath and metal casing were reduced by 10 times in equal proportion in this study. The detailed geometric parameters are given in Fig. 8.

According to the abovementioned geometric parameters of the cement sheath and pore formation method, a porous model of the cement sheath was constructed using a FLAC 3D program developed in this laboratory. To analyze the interaction between the cement sheath and metal casing and their deformation and failure behaviors during the fracturing process, porous reconstruction models of a single-layer cement sheath and double-layer cement sheath were established.

**Figure 9:**
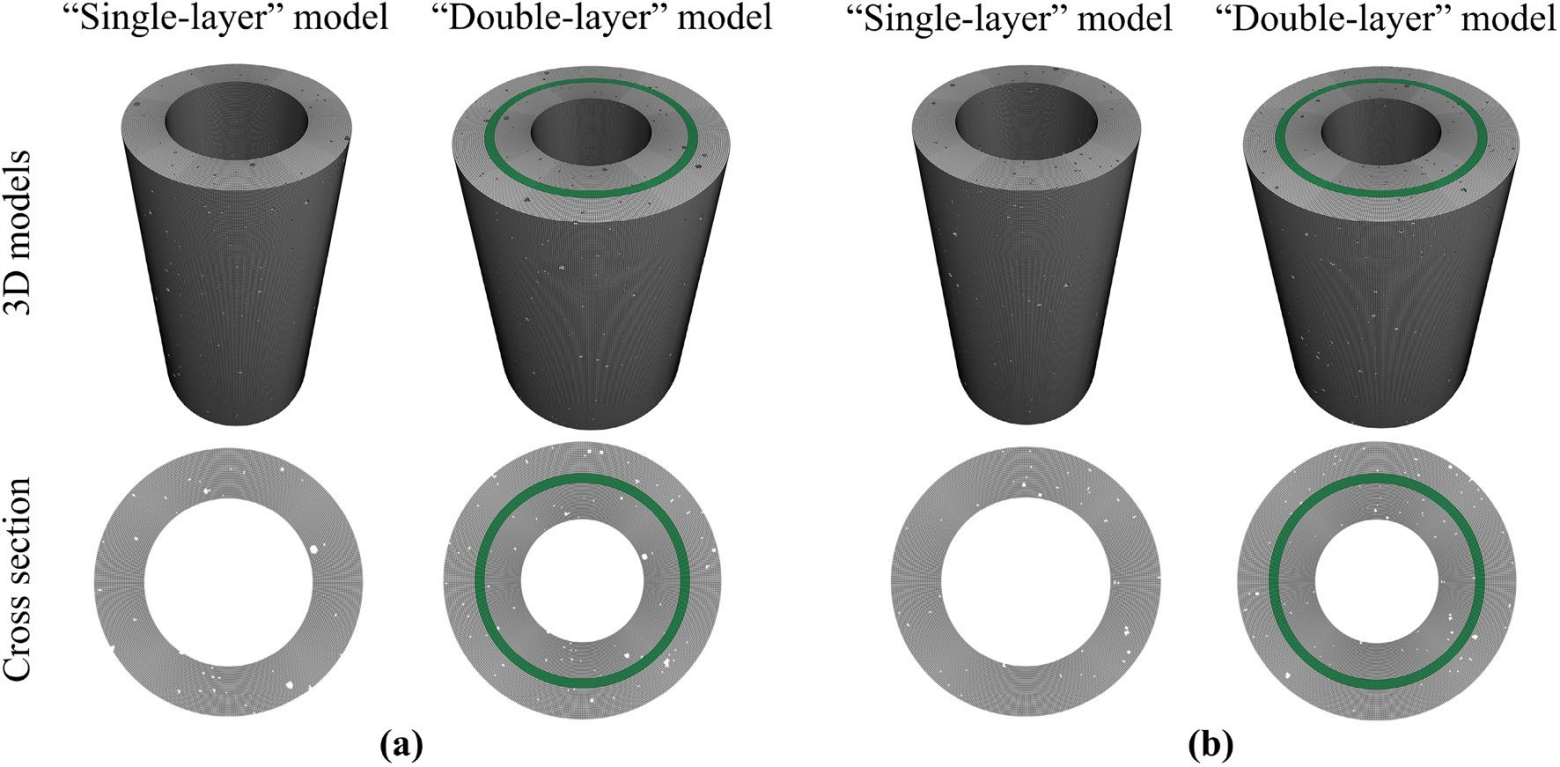
Porous reconstruction models of the cement sheaths. (**a**) Net slurry cement sheath; (**b**) Liquid silicon cement sheath.

Figure 9 shows the porous reconstruction models of the net slurry cement sheath and liquid silicon cement sheath. The single-layer model of the net slurry cement sheath had approximately 3.33 million meshes, and the double-layer model had approximately 6.37 million meshes. The single-layer model of the liquid silicon cement sheath had approximately 4.3 million meshes, and the double-layer model had approximately 7.1 million meshes.

### Boundary conditions of the numerical simulation

According to stress conditions, the cement sheath and metal casing generally do not exhibit axial deformation failure or bending instability. Therefore, vertical displacement constraints were imposed on the upper and lower surfaces of the cement sheath reconstruction model to limit its vertical deformation. An in situ stress of 28 MPa was applied to the surrounding surface of the reconstruction model. Hydraulic pressure was applied to the inner wall of the reconstruction model, which was gradually increased from 0 until the cement sheath failed.

### Failure criterion and constitutive model

The Mohr–Coulomb criterion (Eq. 5) was used to describe the cement sheath failure. The mechanical parameters are listed in Table 2.

5 $$\tau_{f} = c + \sigma \tan \varphi ,$$

where $\tau_{f}$ denotes the shear strength, $c$ is the cohesion force, $\sigma$ is the normal stress on the fractured surface of a material, and $\varphi$ is the internal friction angle.

An isotropic elastic constitutive model was used to characterize the material properties of the metal casing. The volume modulus and shear modulus were set to 167 GPa and 76.9 GPa, respectively.

The isotropic elastic constitutive equation is as follows (Eq. 6):

6 $$\varepsilon = \frac{\sigma }{E},$$

where is $\varepsilon$ the material strain, $\sigma$ is the axial stress, and $E$ is the elastic modulus of a material.

## Results

### Breakdown pressure of cement sheaths

**Figure 10:**
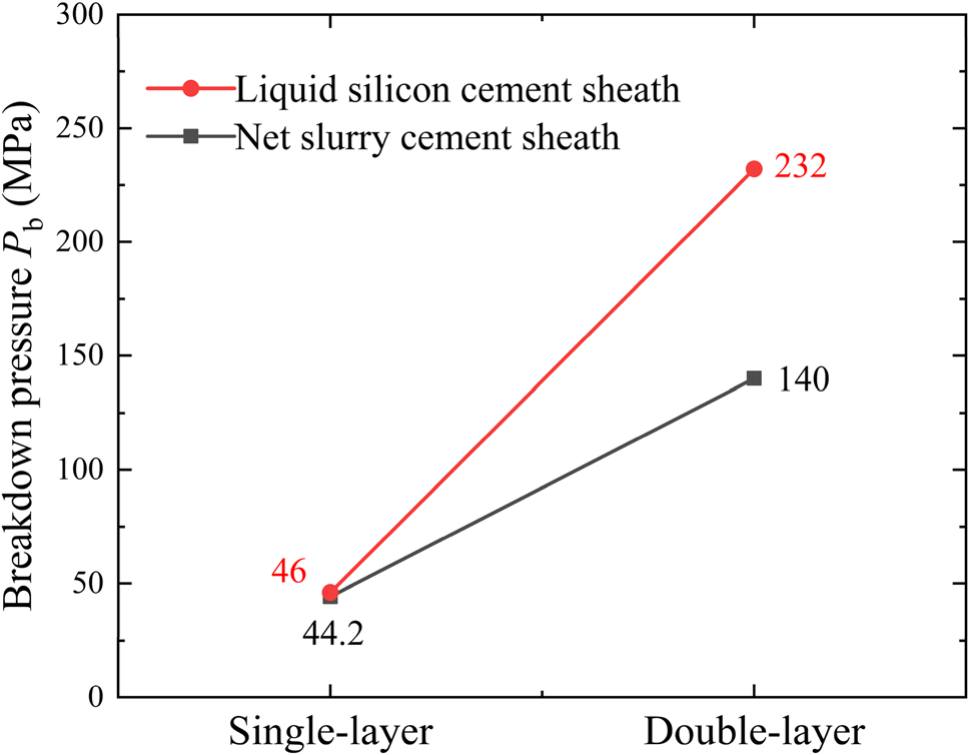
Changes in the breakdown pressure of the cement sheath.

Figure 10 shows the curves of the breakdown pressure of the cement sheath. The results show that there were significant differences in the breakdown pressures between the net slurry cement sheath and the liquid silicon cement sheath as well as between the single-layer and double-layer cement sheaths. The breakdown pressures of the single-layer and double-layer models of the net slurry cement sheath were 44.2 MPa and 140 MPa, respectively, and those of the liquid silicon cement sheath were 46 MPa and 232 MPa, respectively. The breakdown pressure of the liquid silicon cement sheath was obviously greater. The increase in the single-layer model was approximately 4% and that in the double-layer model was approximately 66%. This indicated that the liquid silicon suspension could effectively improve the porous structure inside the cement sheath, reduce the porosity of the cement sheath, and enhance its fracturing resistance. However, the breakdown pressure of the double-layer cement sheath was considerably higher than that of the single-layer sheath. This indicated that the double-layer structure significantly improved the fracturing resistance of the cement sheath. The breakdown pressure of the cement sheath was much greater under the constraints of the double-layer cement sheath and metal casing. In summary, the liquid silicon suspension and the double-layer structure effectively improved fracture resistance and prevented cement sheath failure.

### Plastic failure zones of cement sheaths

**Figure 11:**
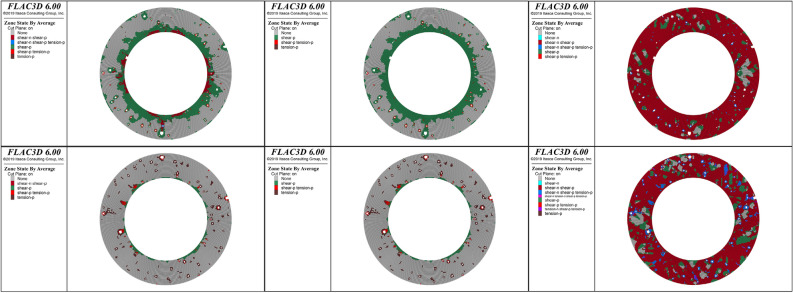
Distribution of plastic zones of the single-layer cement sheath: the rows, from top to bottom, represent the results for the net slurry cement sheath and liquid silicon cement sheath, respectively; the columns, from left to right, represent the results for three loading ratios: 25%, 50%, and 100% of the peak hydraulic pressure.

**Figure 12:**
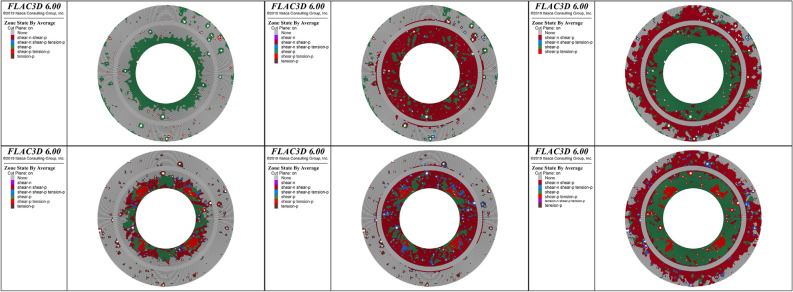
Distribution of plastic zones of the double-layer cement sheath: the rows, from top to bottom, represent the results for the net slurry cement sheath and liquid silicon cement sheath, respectively; the columns, from left to right, represent the results for three loading ratios: 25%, 50%, and 100% of the peak hydraulic pressure.

To analyze the evolution of the plastic failure zones of the cement sheath during fracturing, 25%, 50%, and 100% of the peak hydraulic pressure were selected as loading ratios. The plastic failure characteristics of these three loading ratios were analyzed. Figures 11 and 12 show the distributions of the plastic zones for the three loading ratios of the single-layer and double-layer cement sheaths, respectively. Considering the characteristics of the plane strain, the cross sections of the cement sheath are shown.

For the single-layer cement sheath, Fig. 11 shows that at the initial stage of loading (25% of the peak hydraulic pressure), plastic failure zones appeared in the inner wall and around the pores of the cement sheath. This observation was valid for both the net slurry and liquid silicon cement sheaths. This indicated that cement sheath failure started at the inner wall and pores. The plastic zone area of the net slurry cement sheath was larger than that of the liquid silicon cement sheath. The net slurry cement sheath easily underwent plastic failure under the same hydraulic pressure. For the net slurry cement sheath, the plastic zones were primarily caused by shear failure. For the liquid silicon cement sheath, the plastic zones in the inner wall were caused by shear failure, but those around the pores occurred due to tensile failure owing to the decrease in porosity.

With an increase in hydraulic pressure, the plastic failure zones gradually extended to the outer wall of the cement sheath. This indicated that the failure path of the single-layer cement sheath was from the inner wall to the outer wall. When the hydraulic pressure reached its maximum, the plastic failure zones covered most parts of the cement sheath, and the cement sheath failed completely. The plastic failure zones of the net slurry cement sheath were mainly caused by shear failure, whereas those of the liquid silicon cement sheath were induced by shear failure and tensile failure. The porous structure significantly affected the failure mode of the cement sheath. These pores were likely to cause shear failure in the cement sheath. Cement sheaths with low porosity underwent tensile and shear failures, whereas cement sheaths with high porosity mainly underwent shear failure.

**Figure 13:**
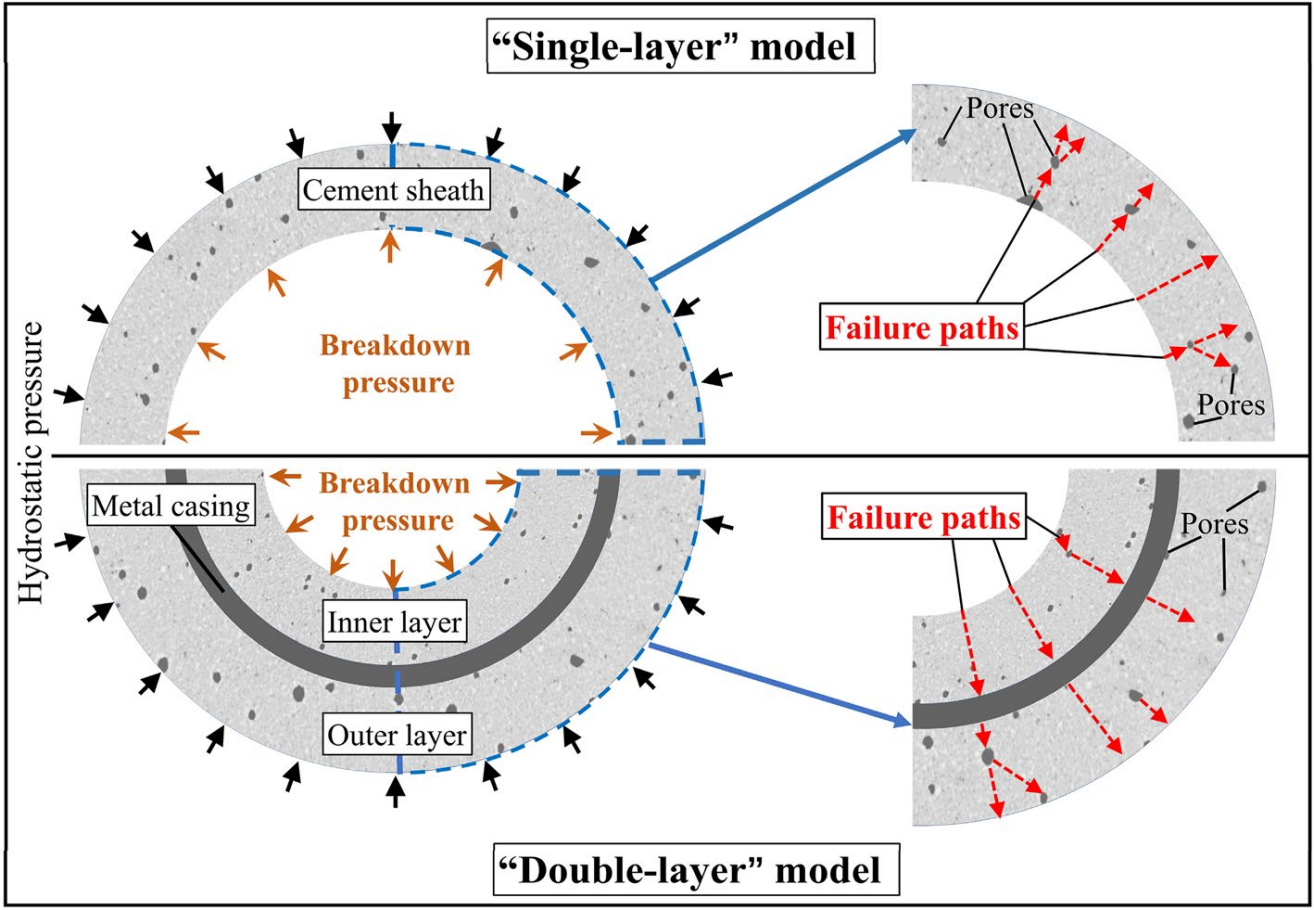
Failure paths of cement sheaths.

For the double-layer cement sheath, the interaction between the cement sheath and metal casing resulted in a significant change in the failure mode compared with the single-layer cement sheath (Fig. 12). At the beginning of loading, most of the plastic failure zones appeared in the inner layer of the cement sheath, and the remainder appeared around the pores in the outer layer of the cement sheath. The plastic zones of the net slurry cement sheath were mostly caused by shear failure, whereas the plastic zones of the liquid silicon cement sheath were caused by both shear and tensile failures. The change in porosity resulted in a completely different failure mode around the pores. That is, the failure mode around the pores of the net slurry cement sheath was complete shear failure, whereas that of the liquid silicon cement sheath was complete tensile failure. When the hydraulic pressure reached the maximum, the inner layer cement sheath completely failed, and most parts of the outer layer of the cement sheath underwent plastic failure, whereas the metal casing remained intact. This indicated that the double-layer structure effectively protected the metal casing and prevented metal casing failure. Moreover, the double-layer structure of the cement sheath strengthened the effect of the pores on the plastic failure zone of the cement sheath. In contrast to the single-layer liquid silicon cement sheath, the plastic zones induced by the tensile failure of the double-layer liquid silicon cement sheath decreased, and the plastic zones induced by the shear failure increased. Figure 13 shows a schematic diagram of the failure paths of the cement sheaths.

### Radial plane deformation of cement sheaths

**Figure 14:**
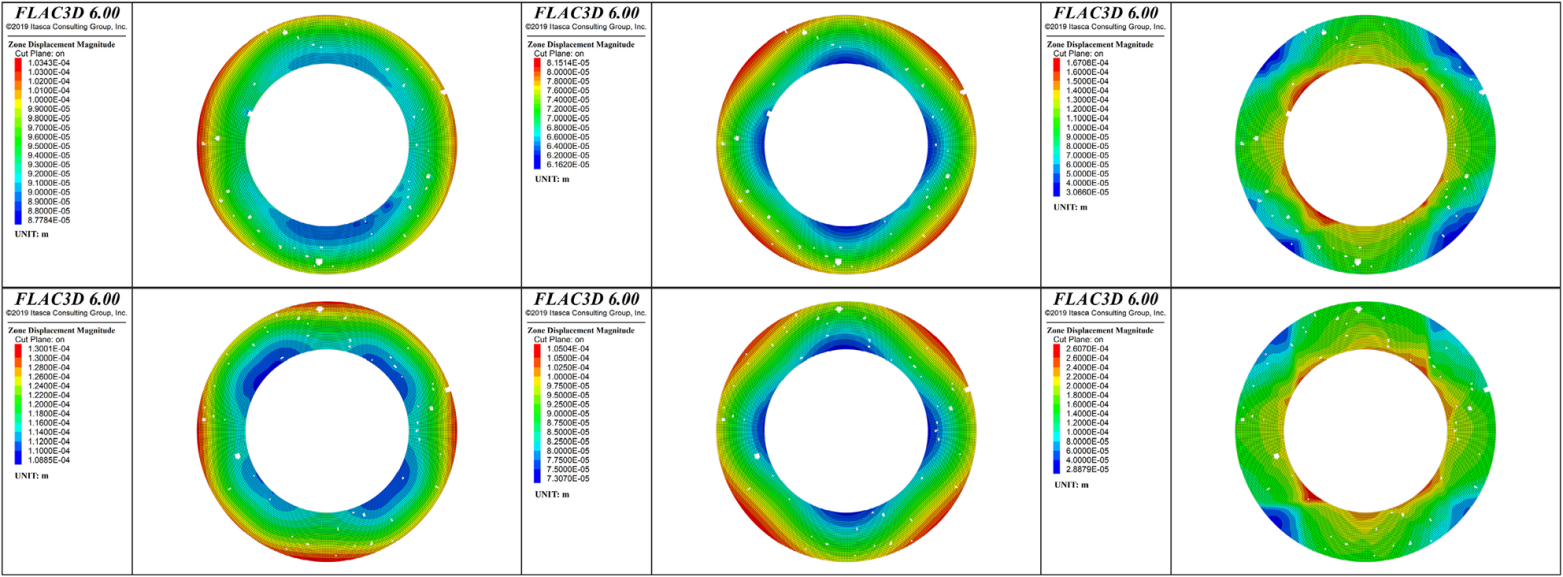
Radial plane deformation of the single-layer cement sheath: the rows, from top to bottom, represent the results for the net slurry cement sheath and liquid silicon cement sheath, respectively; the columns, from left to right, represent the results for three loading ratios: 25%, 50%, and 100% of the peak hydraulic pressure.

**Figure 15:**
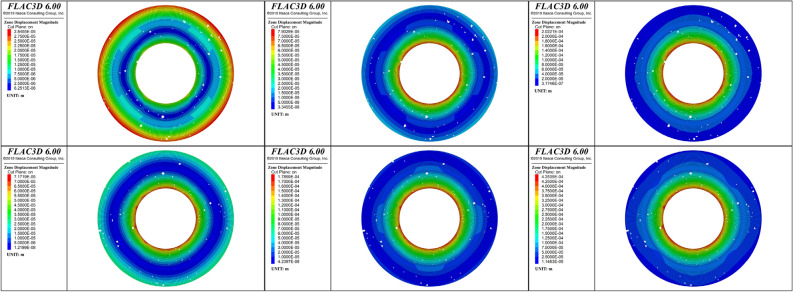
Radial plane deformation of the double-layer cement sheath: the rows, from top to bottom, represent the results for the net slurry cement sheath and liquid silicon cement sheath, respectively; the columns, from left to right, represent the results for three loading ratios: 25%, 50%, and 100% of the peak hydraulic pressure.

To analyze the deformation behaviors of the cement sheaths during fracturing, radial plane displacement images of the cross sections of the cement sheath with the three loading ratios were obtained. Figures 14 and 15 show the results for the single-layer and double-layer cement sheaths, respectively.

According to Fig. 14, the porous structure significantly affected the radial deformation of the single-layer cement sheath. Owing to the effect of the pores, the radial displacement distribution of the single-layer cement sheath was not symmetrical. At the initial stage of loading, under the action of in situ stress and hydraulic pressure, the cement sheath underwent outward displacement along the radial direction, causing the cement sheath as a whole to expand outward. However, the radial displacement of the cement sheath differed along the thickness, and that of the outer wall was greater than that of the inner wall. This indicated that the in situ stress played a major role in the radial deformation of the cement sheath at the initial stage of loading. As the hydraulic pressure was increased, the radial displacement of the inner wall of the cement sheath increased rapidly. When the hydraulic pressure reached its maximum, the radial displacement of the inner wall of the cement sheath exceeded that of the outer wall, and the maximum value appeared at the inner wall of the cement sheath. Hydraulic pressure rather than in situ stress was the main factor controlling the radial deformation of the cement sheath. This also verified that the failure path of the single-layer cement sheath was from the inner wall to the outer wall. The maximum radial displacements of the net slurry and liquid silicon cement sheaths were 167 μm and 260 μm, respectively. The maximum value of the radial displacement of the liquid silicon cement sheath was obviously greater than that of the net slurry cement sheath. This indicated that the liquid silicon suspension effectively improved the deformation ability of the cement sheath in addition to its fracture resistance.

The deformation characteristics of the double-layer cement sheath differed considerably from those of the single-layer cement sheath. Because the casing was stiffer than the cement sheath, the minimum radial displacement occurred at the metal casing at the initial stage of loading, and the maximum appeared in the inner and outer layers of the cement sheath. The magnitude of the displacement of the inner layer of the cement sheath did not differ from that of its outer layer. With a gradual increase in hydraulic pressure, the radial displacement of the inner layer of the cement sheath increased rapidly, whereas that of its outer layer increased slowly. When the hydraulic pressure reached its maximum, the radial displacement was maximum in the inner layer of the cement sheath, and the radial displacement gradually decreased along the radial direction of the cement sheath outward. Compared with the single-layer cement sheath, the distribution of the radial displacement of the double-layer cement sheath was more symmetrical and showed an annular distribution. The double-layer structure weakened the effect of pores on the deformation of the cement sheath. The maximum radial displacements of the net slurry and liquid silicon cement sheaths were 202 μm and 425 μm, respectively. The maximum value of the radial displacement of the liquid silicon cement sheath was larger than that of the net slurry cement sheath, which was consistent with the conclusion about the single-layer cement sheath. However, the maximum radial displacement of the double-layer cement sheath was considerably larger than that of the single-layer cement sheath, indicating that the double-layer structure also effectively improved the deformation ability of the cement sheath.

### Stress characteristics of cement sheaths

**Figure 16:**
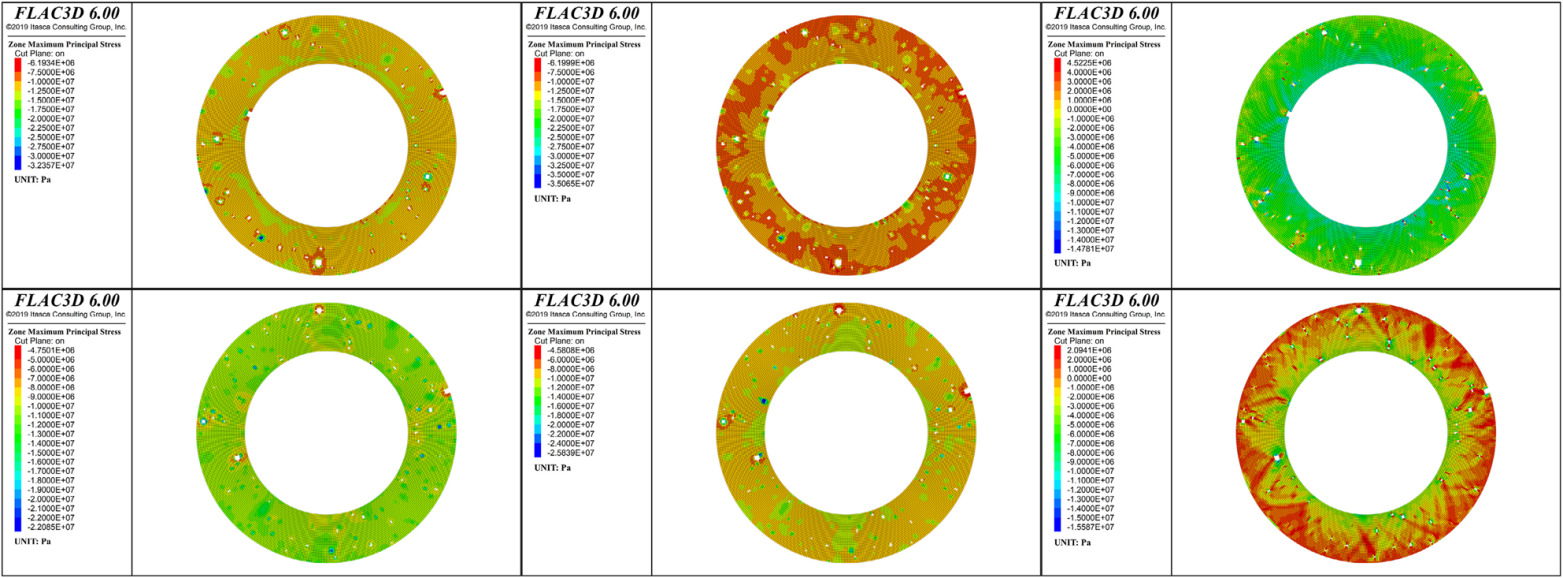
Major principal stress of the single-layer cement sheath: the rows, from top to bottom, represent the results for the net slurry cement sheath and liquid silicon cement sheath, respectively; the columns, from left to right, represent the results for three loading ratios: 25%, 50%, and 100% of the peak hydraulic pressure.

**Figure 17:**
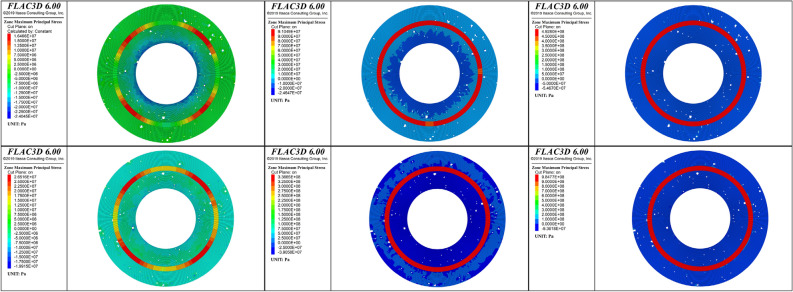
Major principal stress of the double-layer cement sheath: the rows, from top to bottom, represent the results for the net slurry cement sheath and liquid silicon cement sheath, respectively; the columns, from left to right, represent the results for three loading ratios: 25%, 50%, and 100% of the peak hydraulic pressure.

The influences of pores and the single-double layer structure of the sheath on the stress distribution and the internal failure mechanism of the cement sheath were analyzed. To this end, the major principal stress evolution of the cross sections of the cement sheathes were analyzed during fracturing. Figures 16 and 17 show the major principal stresses of the single-layer and double-layer cement sheaths for the three loading ratios, respectively.

At the early stage of loading, the major principal stress of the single-layer cement sheath was completely in a state of compressive stress. Because the pore sizes in the liquid silicon cement sheath were smaller than those in the net slurry cement sheath, its stress concentration was more obvious, and the magnitude of the compressive stress value was greater. As the hydraulic pressure was increased, the major principal stress of the cement sheath gradually increased. When the hydraulic pressure reached its peak load, the stress distribution in the cement sheath changed significantly. Both compressive and tensile stresses appeared in the cement sheath. The compressive stress zones were significantly larger than the tensile stress zones in the net slurry cement sheath, whereas there was a slight difference between them in the liquid silicon cement sheath. In other words, the net slurry cement sheath was still dominated by compressive stress, whereas the liquid silicon cement sheath was dominated by both compressive and tensile stresses. Therefore, the net slurry cement sheath mainly underwent compressive-shear failure, whereas the liquid silicon cement sheath underwent shear and tensile failures. This indicated that the porous structure had a significant influence on the failure mode of the cement sheath from a stress perspective.

**Figure 18:**
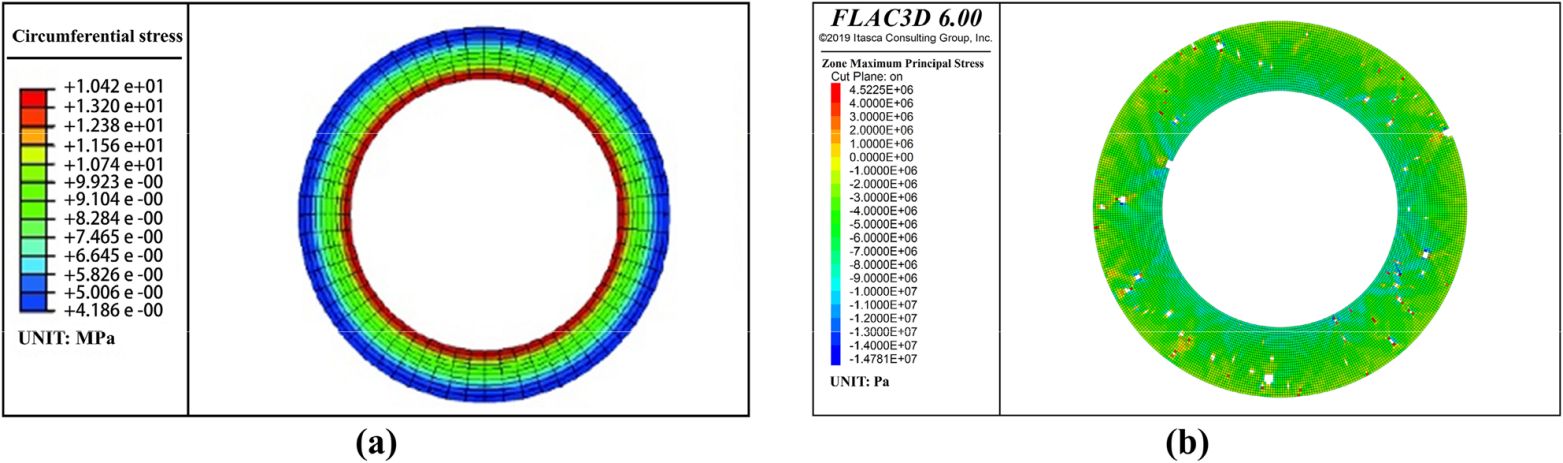
Stress distribution of the cross section of the single-layer cement sheath^48^. (**a**) Net slurry cement sheath; (**b**) Liquid silicon cement sheath.

The influence of pores on the stress distribution of the single-layer cement sheath was analyzed further. To this end, the stress results for the porous cement sheath were compared with those for a nonporous cement sheath obtained by other researchers^48^. The results are shown in Fig. 18.

In contrast, the major principal stress of the cross section of the nonporous cement sheath was in a state of complete tensile stress. The magnitude of the stress of the inner wall was greater than that of the outer wall of the cement sheath. This indicated that the failure mode of the nonporous cement sheath was mainly tensile failure, and the failure path was from the inside to the outside. However, the porous structure significantly changed the stress state of the single-layer cement sheath, which transformed from only tensile stress to both tensile and shear stresses. Accordingly, the failure mode of the porous cement sheath changed from tensile failure to a combination of tensile and shear failures. The larger the porosity was, the larger the proportion of shear failure. This indicated that the pores were tended to cause shear failure of the cement sheath. Notably, the porous structure did not change the failure path of the cement sheath. Regardless of the porosity, the failure path of the porous cement sheath was from the inside to the outside.

The major principal stress distributions differed significantly for the double-layer and the single-layer cement sheaths. Compressive and tensile stresses appeared in the cement sheath during the early stage of loading. Tensile stress mainly occurred in the metal casing, and compressive stress appeared in the cement sheath. The magnitude of the compressive stress was greater for the inner layer than the outer layer of the cement sheath. This indicated that the double-layer cement sheath failed first at the inner layer of the cement sheath. This conclusion was consistent with that obtained from the analysis of the plastic failure zone. With an increase in hydraulic pressure, the compressive stress of the inner layer of the cement sheath gradually reached the maximum value, whereas the compressive stress value was always less for the outer layer than the inner layer. When the hydraulic pressure reached the maximum, the major principal stresses of both the net slurry and liquid silicon cement sheaths completely entered the compressive stress state. The major principal stress of the metal casing remained in a state of tensile stress. Compared with the single-layer cement sheath, the stress state of the double-layer net slurry cement sheath changed slightly, and the major principal stress was still compressive stress. However, the stress state of the double-layer liquid silicon cement sheath was very different, from a state of both tensile stress and compressive stress to a state of only compressive stress. The double-layer structure strengthened the influences of the porous structure on the stress distribution and failure mode of the cement sheath, which led to a single shear failure in the cement sheath with low porosity.

## Conclusions

In this study, porous reconstruction models of single- and double-layer cement sheaths were established. Based on the reconstruction models, the deformation failures of the cement sheaths were studied under in situ stress and hydraulic pressure. The effects of pores and single-double layer structure on the deformation failure of the cement sheaths were analyzed in terms of breakdown pressures, plastic zones, deformations, and stress distributions. The results revealed the failure mechanisms of the porous cement sheath. The main conclusions were as follows:

1. The spatial locations of the pores in the net slurry cement sheath and liquid silicon cement sheath approximately satisfied uniform distributions along the circumferential direction, and the pore radii followed Gaussian distributions. The pore size and porosity of the liquid silicon cement sheath were smaller than those of the net slurry cement sheath. Adding liquid silicon suspension to a mixture of materials effectively reduced the pore size and porosity of the cement sheath and increased its compactness.
2. The smaller the porosity of the cement sheath was, the higher the breakdown pressure. The breakdown pressure was significantly higher for the double-layer cement sheath than the single-layer cement sheath. The double-layer structure and liquid silicon suspension can effectively improve the fracture resistance and prevent damage to the cement sheath.
3. The failure path of the cement sheath occurred from the inner wall and pore periphery to the outer wall, and the porous structure slightly influenced this failure path. However, the porous structure significantly influenced the failure mode of the cement sheath. The liquid silicon cement sheath with little porosity underwent tensile and shear failures, whereas the net slurry cement sheath with much porosity underwent mainly shear failure. Under the same hydraulic pressure, the more porous cement sheath was more likely to undergo plastic failure. However, the double-layer cement sheath effectively prevented failure of the metal casing. Moreover, the double-layer structure enhanced the effect of pores in the plastic failure zone of the less porous cement sheath, and the shear plastic failure zone became larger.
4. The porous structure significantly affected the radial deformation of the cement sheath. When the cement sheath was less porous, it underwent larger radial displacement. Owing to the influence of pores, the distribution of radial displacement of the single-layer cement sheath was asymmetrical, and the radial displacement of the outer wall was less than that of the inner wall. The double-layer structure weakened the effect of pores on the deformation of the cement sheath such that the radial displacement of the double-layer cement sheath has a certain symmetry and showed a circular distribution. The radial displacement was significantly larger for the double-layer cement sheath than the single-layer cement sheath. Both the liquid silicon suspension and double-layer structure effectively improved the deformation ability of the cement sheath.

The results of this study provide a basis for an in-depth understanding of the effects and mechanisms of the influence of porosity on the breakdown pressures, deformations, stress distributions, and failure modes of cement sheaths. Previous engineering practice has shown that shear damage is the main cause of cement sheath failure during exploitation of unconventional energy sources. The results showed that the use of the double-layer structure of the cement sheath and the reduction in cement sheath porosity effectively prevented shear damage of the cement sheath. The study provides a theoretical basis and concept for the prevention of shear damage of cement rings in engineering applications in terms of both the structural and porosity of cement sheaths. Notably, this study considers a cement sheath with one material ratio as the research object, and it will be necessary to verify whether the conclusions are applicable to cement sheaths with other material ratios. However, the reconstruction method of the porous model of the cement sheath presented in this paper provides an effective approach to study the failure mechanisms of cement sheaths with other material ratios.

## Data Availability

The datasets generated and/or analyzed during the current study are available from the corresponding author on reasonable request.
